# A broad wastewater screening and clinical data surveillance for virus-related diseases in the metropolitan Detroit area in Michigan

**DOI:** 10.1186/s40246-024-00581-0

**Published:** 2024-02-06

**Authors:** Yabing Li, Brijen Miyani, Russell A. Faust, Randy E. David, Irene Xagoraraki

**Affiliations:** 1 Department of Civil and Environmental Engineering, Michigan State University, 1449 Engineering Research Ct, East Lansing, MI 48823 USA; 2 Oakland County Health Division, 1200 Telegraph Rd, Pontiac, MI 48341 USA; 3 School of Medicine, Wayne State University, Detroit, MI 48282 USA

**Keywords:** Wastewater surveillance, Metagenomics, Human virus, Public health, COVID-19 outbreak, Viral epidemiology, Predictive intelligence

## Abstract

**Background:**

Periodic bioinformatics-based screening of wastewater for assessing the diversity of potential human viral pathogens circulating in a given community may help to identify novel or potentially emerging infectious diseases. Any identified contigs related to novel or emerging viruses should be confirmed with targeted wastewater and clinical testing.

**Results:**

During the COVID-19 pandemic, untreated wastewater samples were collected for a 1-year period from the Great Lakes Water Authority Wastewater Treatment Facility in Detroit, MI, USA, and viral population diversity from both centralized interceptor sites and localized neighborhood sewersheds was investigated. Clinical cases of the diseases caused by human viruses were tabulated and compared with data from viral wastewater monitoring. In addition to *Betacoronavirus*, comparison using assembled contigs against a custom Swiss-Prot human virus database indicated the potential prevalence of other pathogenic virus genera, including: *Orthopoxvirus*, *Rhadinovirus, Parapoxvirus*, *Varicellovirus, Hepatovirus, Simplexvirus, Bocaparvovirus, Molluscipoxvirus, Parechovirus, Roseolovirus, Lymphocryptovirus, Alphavirus, Spumavirus, Lentivirus, Deltaretrovirus, Enterovirus, Kobuvirus, Gammaretrovirus, Cardiovirus, Erythroparvovirus, Salivirus, Rubivirus, Orthohepevirus, Cytomegalovirus, Norovirus,* and *Mamastrovirus*. Four nearly complete genomes were recovered from the *Astrovirus, Enterovirus, Norovirus and Betapolyomavirus* genera and viral species were identified.

**Conclusions:**

The presented findings in wastewater samples are primarily at the genus level and can serve as a preliminary “screening” tool that may serve as indication to initiate further testing for the confirmation of the presence of species that may be associated with human disease. Integrating innovative environmental microbiology technologies like metagenomic sequencing with viral epidemiology offers a significant opportunity to improve the monitoring of, and predictive intelligence for, pathogenic viruses, using wastewater.

**Supplementary Information:**

The online version contains supplementary material available at 10.1186/s40246-024-00581-0.

## Background

In combination with classic epidemiological methods, wastewater surveillance has been repeatedly validated as a useful method for predicting viral disease outbreaks in communities with centralized wastewater collection systems. Wastewater surveillance of severe acute respiratory syndrome coronavirus 2 (SARS-CoV-2) has occurred globally in an effort to combat the COVID-19 pandemic, demonstrating the importance of applied wastewater surveillance in understanding virus transmission dynamics and for serving as an early warning system [1–9]. Wastewater surveillance approaches that extend beyond the surveillance of confirmed viral diseases in a community and extend to multiple reportable and non-reportable virus-related diseases are needed.

Hundreds of viral species can infect humans with novel species or subspecies variants continuing to be identified [10, 11] and enteric, respiratory, bloodborne, and vector-borne viruses have all been confirmed to be detectable in wastewater samples [12–17]. Whether officially reportable or non-reportable to public health practitioners, or whether, endemic, emerging, or novel, viral threats are circulating within communities. Wastewater surveillance tools that are both practical in application and capable of accurately and efficiently identifying a diversity of human viral pathogens in varied environments, are urgently needed.

Identifying bacterial population diversity using sequencing and metagenomics is relatively straightforward, as bacteria contain a shared gene, 16sRNA, that can be sequenced for phylogenetic analysis of bacteria. In contrast, viruses do not unilaterally share any conserved gene, making it difficult to calculate indices of viral population genetic diversity. Random amplification and high-throughput shotgun sequencing, followed by appropriate bioinformatic analysis can largely circumvent these limitations, presenting an opportunity to expand the translatability of wastewater surveillance methodologies. Nonetheless, there are significant challenges in metagenomic-enabled wastewater surveillance. For example, a relatively lower abundance of human viruses [10, 18, 19] compared to the bacterial community in wastewater samples poses challenges for sequencing and bioinformatic analyses; therefore, proper sample concentration is critical. Other challenges, like computational resources, human capital requirements, and bioinformatic analysis training, may limit the adoption of high-throughput shotgun sequencing methods. In this paper, we present a bioinformatics-based screening tool that focuses on viral population diversity identification. The screening tool is validated in wastewater samples collected from the Detroit metropolitan area during the COVID-19 pandemic, and the results reveal that, in addition to coronaviruses, multiple viral genera are present in the tested community wastewater.

Within the Detroit metropolitan area, wastewater surveillance has been applied to detect multiple human virus occurrences [12, 16, 17, 20]. Since the onset of the COVID-19 epidemic in the Detroit metropolitan area, a wastewater surveillance program was focused on SARS-CoV-2 detection and has since shown to be an important tool in (1) providing early warnings of disease surges [6, 21], (2) dissecting the spatial distribution of SARS-CoV-2 concentrations across a large geographic area in communities with diverse demographic characteristics [7], and (3) developing straightforward methods designed to assist public health officials in mounting a timely and appropriate response [22]. In this study, we investigate human virus diversity beyond coronaviruses. We collected a total of 48 untreated “grab” wastewater samples collected from interceptors at the wastewater treatment facility, and manholes in neighborhoods from the service area of three interceptors. Human virus compositions at the genus level were analyzed and discussed. Classification of four viral pathogens was performed at the genotype level using the nearly complete draft genomes recovered. Clinical case data of the diseases associated with the studied viruses during the sampling year were collected and compared with the data from wastewater samples. Interpretation of the human virus composition in wastewater at the genus level and recovery of genomes using bioinformatics methods contribute to our understanding of the infectious diseases circulating in metropolitan Detroit community. Limitations of the untargeted sequencing approach and the optimization possibilities were discussed.

## Methods

### Study area and sample collection

**Fig. 1:**
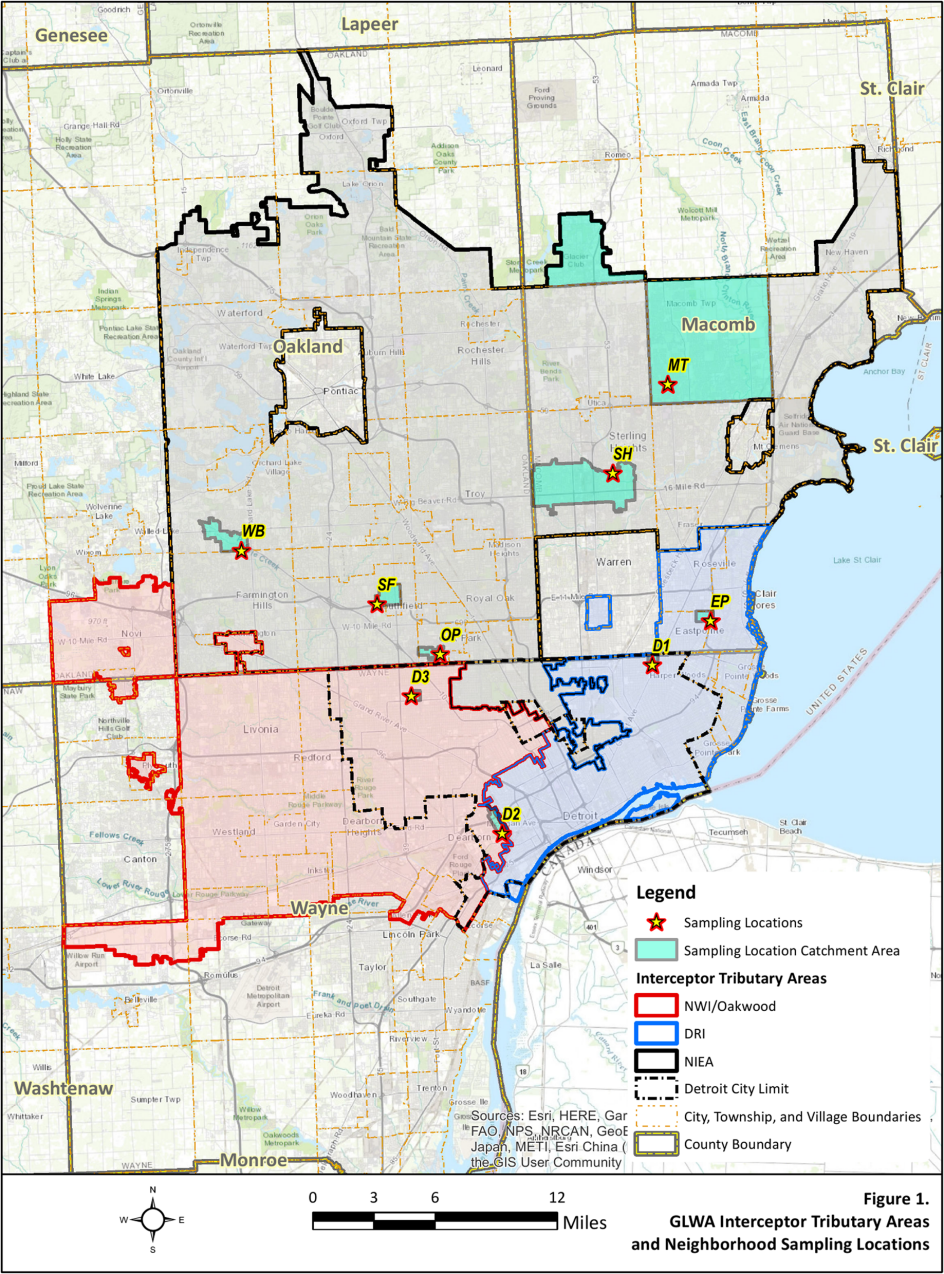
Service area of the three interceptors of the Water Resource Recovery Facility (WRRF). The yellow stars indicate the nine neighborhood locations from three counties that WRRF serves

The Water Resource Recovery Facility (WRRF) is the wastewater system of the Great Lakes Water Authority (GLWA) in Detroit, Michigan. The WRRF is the largest single-site wastewater treatment facility in North America and serves the largest city in Michigan, as well as the three most populous counties in Michigan: Wayne, Oakland, and Macomb [23]. The facility receives wastewater via three main interceptors, including the Detroit River Interceptor (DRI), the North Interceptor-East Arm (NI-EA), and the Oakwood-Northwest-Wayne County Interceptor (O-NWI). Combined, the three interceptors serve approximately 492,000 (DRI), 1,482,000 (NI-EA), and 840,600 (O-NWI) individuals, based on 2020 population estimates provided by the Southeast Michigan Council of Governments. The WRRF system collects and treats stormwater along with residential, industrial, and commercial waste, depending on service area. There were seven sample collection events across the three interceptors, for a total of 21 interceptor samples. Three sampling events were occurred from Wayne, Oakland, and Macomb Counties at the neighborhood sewershed level, resulting in a total of 27 neighborhood samples. Sampling locations in these three counties were selected to ensure that data collectively represented community demographics [7]. Sampling site locations and the service area of the three interceptors are shown in Fig. 1. Catchment area population characteristics and sampling dates are shown in Table 1.

**Table 1:** Catchment area population characteristics and sampling dates

Interceptor sites		
Sample site	Corresponding population	Sampling dates
O-NWI	840,600	8/11/20, 2/2/21, 2/22/21, 6/28/21, 10/4/21, 12/27/21, 1/17/22
NI-EA	1,482,000	
DRI	492,000	

Neighborhood sites				
County	Sample site	Area (km^2^)	Corresponding population	Sampling dates
Macomb	Eastpointe (EP)	1.13	2400	2/4/21, 3/18/21, 10/7/21
	Macomb Township (MT)	90.1	99,970	
	Sterling Heights (SH)	25.3	37,560	
Wayne	Detroit 1 (D1)	0.55	1690	
	Detroit 2 (D2)	1.50	5190	
	Detroit 3 (D3)	0.51	1300	
Oakland	Southfield (SF)	2.90	3080	
	West Bloomfield (WB)	4.93	5800	
	Oak Park (OP)	1.16	2270	

### Collection and virus concentration analysis of wastewater sample

Viruses were collected and isolated from wastewater using electropositive NanoCeram column filters (Argonide, Sanford, FL, USA) based on an US Environmental Protection Agency (EPA) protocol [20, 21, 24]. Depending on the quantity of suspended solids in the wastewater sample, approximately 20 to 50 L of raw wastewater was passed through NanoCeram electropositive cartridge filters at a rate not more than 11 L/min. Flow meter readings were recorded at the commencement and termination of each sampling event, so as to measure the total volume of raw wastewater that passed through the filter. The filters containing viruses were placed in separated, sealed plastic bags on ice, and transported to the Environmental Virology Laboratory at Michigan State University in East Lansing, MI, for analysis within 48 h.

Electropositive NanoCeram column filters were eluted with 1 L beef extract (MilliporeSigma, Massachusetts, USA) solution (prepared before elution) for 2 min. After, pH of the beef solution was adjusted to 3.5 ± 0.1, then flocculated for 30 min before centrifugation at 2500*g* (at 4 °C) for 15 min. The supernatant was discarded, and pellets were then resuspended in 30 mL of sodium phosphate (at 0.15 M). The pH of the resuspended solution was adjusted to a range of 9.0–9.5. A second round of centrifugation was then carried out at 7000*g* (at 4 °C) for 10 min. The supernatant was collected and adjusted to a pH of approximately 7.25. Filtration was performed on the samples with 0.45 μm and 0.22 μm syringe filters to eliminate the contamination of bacteria with large sizes. The final filtered solution was then aliquoted into multiple 2.0 mL cryogenic vials (Corning®, New York, USA) and stored at − 80 °C until nucleic acid extraction was performed.

### Sequencing

#### Extraction of nucleic acids and random amplification

Viral nucleic acids were extracted using QIAGEN QIAamp Viral RNA kits (QIAGEN, Hilden, Germany), following the manufacturer’s protocol with the volume of final eluting reagent (buffer AVE) modified from 60 to 140 µL [6, 7, 16]. To ensure enough sample for the final metagenomic library, extracts of duplicate samples were pooled together. A random-primer protocol developed to identify viral pathogens was applied to perform the amplification [25, 26]. Primer-A (5′-GTTTCCCAGTCACGATCNNNNNNNNN) was used to do the RNA reverse-transcription, and second-strand DNA synthesis was carried out using Sequenase (version 2.0 DNA Polymerase, Thermo Fisher Scientific). The subsequent PCR amplification of 40 cycles was finished with primer-B (5′-GTTTCCCAGTCACGATC) [12, 25, 26].

#### Next generation sequencing

Viral cDNA from the wastewater samples (n = 48) was sent to the Michigan State University’s Research Technology Support Facility's Genomics Core for library preparation and sequencing. Details of library preparation and sequencing are provided in Additional file 1: S1. Quality of the raw reads was assessed using FastQC [27] analysis. Quality scores for more than 88% of both R1 and R2 reads in every sample were better than 30. A total of 5.91 billion reads were obtained for the 48 samples and the average number of reads for each sample was 104 million. The 48 samples had an average yield of 31.1 gigabytes (GB).

### Bioinformatic analysis

#### Trimming, assembling, and taxonomic alignment

Adapters and low-quality reads were trimmed using Trimmomatic (v. 0.39, parameters: phred33 TruSeq3-PE.fa:2:30:10 LEADING:3 TRAILING:3 SLIDINGWINDOW:4:15 MINLEG:35) [28]. Trimmed reads were then aligned against the National Center for Biotechnology Information’s (NCBI) BLAST non-redundant database using Kaiju (v. 1.9.0), to determine the proportions of viral reads in the samples. A sensitive run mode, “Greedy” was used, and the cutoff for *E* value was set to 10^–3^ [29].

To achieve substantial gains in taxonomic mapping, long contiguous sequences (contigs) generated by the assembly process were used to identify viral and human viral composition [30]. To identify the virus composition in wastewater samples, the assembled contigs were aligned against the NCBI’s RefSeq virus database (retrieved on December 1, 2022) with DIAMOND BLASTx, using a maximum *E* value of 10^–3^ [12, 31, 32]. In order to improve the discovery of human viruses, reduce ambiguity, and decrease the chance of false negative hits [12], the assembled contigs were also aligned against a custom Swiss-Prot human virus protein database using BLASTx, using a maximum *E* value of 10^–5^. Details on how we customized the Swiss-Prot human virus protein database are provided in Additional file 1: S2. Virus compositions at the family level and human viruses at the genus level were further obtained using MEGAN software, Community Edition (v. 6.22.2) [33].

#### Quality check for human virus contigs and phylogenetic analysis of near-complete contigs

Quality and completeness of the human virus-related contigs identified in the wastewater samples were estimated using CheckV [34], a command-line pipeline used to identify closest genomes and host contamination for integrated proviruses, and ultimately, estimate completeness of genome fragments. All of the human virus-related contigs were assigned to one of the quality tiers (High-quality, Medium-quality, Low-quality) or were determined to be of Undetermined quality, based on genome completeness, host contamination, and the predicted closest genomes. Four representative, near-complete contigs were found, with high genome-wide sequence similarities to their reference genomes, according to ViPTree (v. 3.3) [35]. Genes and their positions were predicted with GeneMarkS [36]. Genome structures of the four near-complete draft genomes and their closest reference genomes were visualized using the Proksee platform [37].

**Fig. 2:**
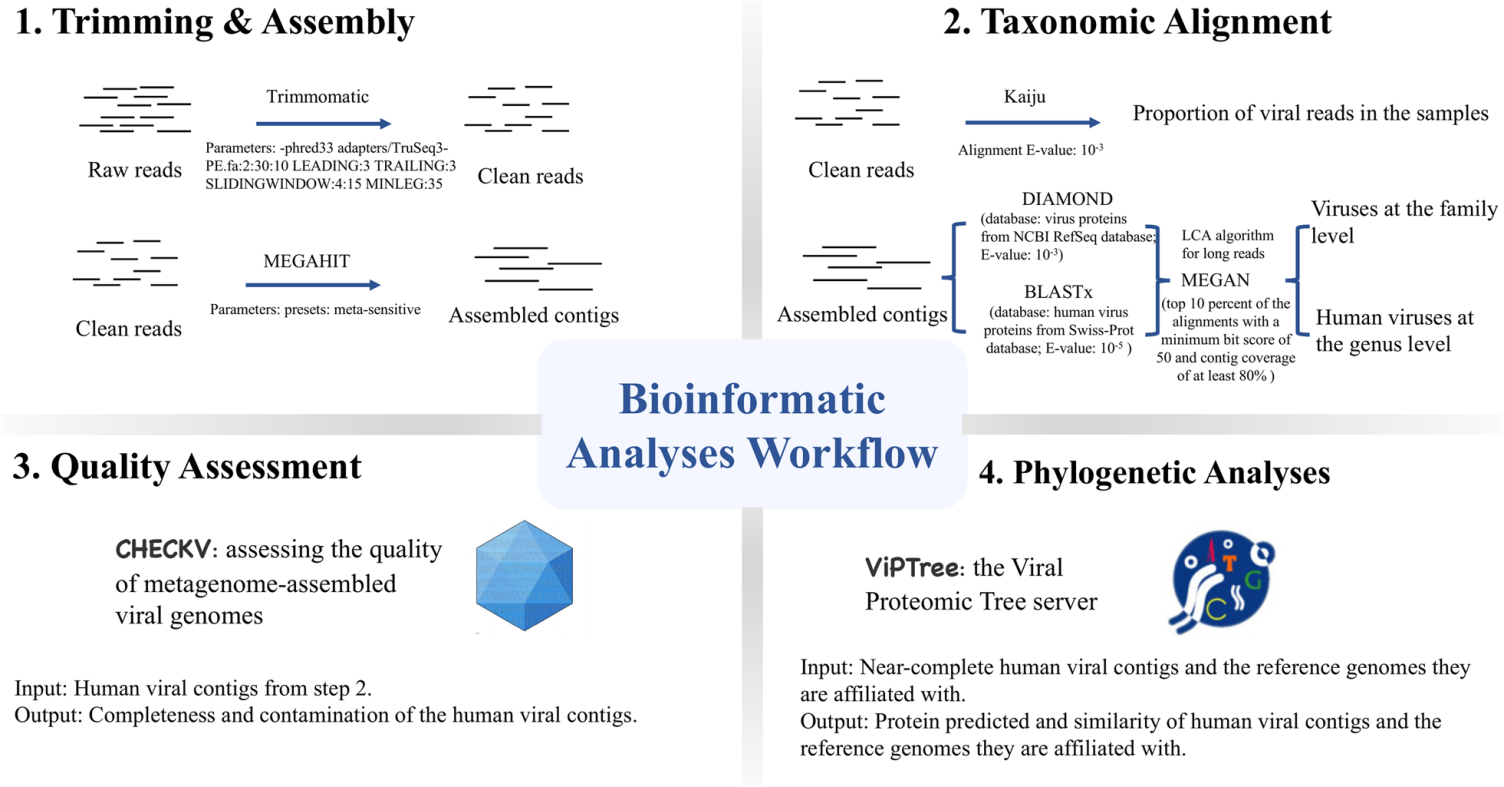
A schematic workflow for identifying human virus occurrence in wastewater using metagenomics-enabled surveillance

A bioinformatic workflow schematic for identifying human virus occurrence in wastewater samples with a metagenomics-enabled surveillance approach is shown in Fig. 2. Parameters applied in the bioinformatic process are the same as indicated in our previous work [38] and are elaborated in Additional file 1: Table S1.

#### Statistical analysis and data visualization

After comparing against the NCBI non-redundant database using kaiju, percentages of reads affiliated with viruses, bacteria, archaea, and unknown reads were calculated by using the number of viral, bacterial, archaeal, and unknown reads divided by the total reads in the sample. As for the proportions of each human viral family and genus, data were normalized to the human viral community. Data were organized and the proportions of each human virus were calculated using Excel (Microsoft, Redmond, WA). Statistical analyses including the Wilcoxon mean value test and non-metric multidimensional scaling (NMDS) were performed using RStudio [39]. Illustrations including pie charts, violin plots, bubble plots, heatmaps, and plots for NMDS were created using RStudio. Packages including “dplyr,” “ggplot2,” “scatterpie,” “vegan,” “BiodiversityR,” and “tidyverse” were used in the statistical analyses and data visualization process. Genome visualizations generated by the Proksee platform were organized and customized using the vector graphics editor, Inkscape (v. 1.2.2).

## Results

### Outputs from the sequencing of 21 interceptor and 27 neighborhood wastewater samples

**Fig. 3:**
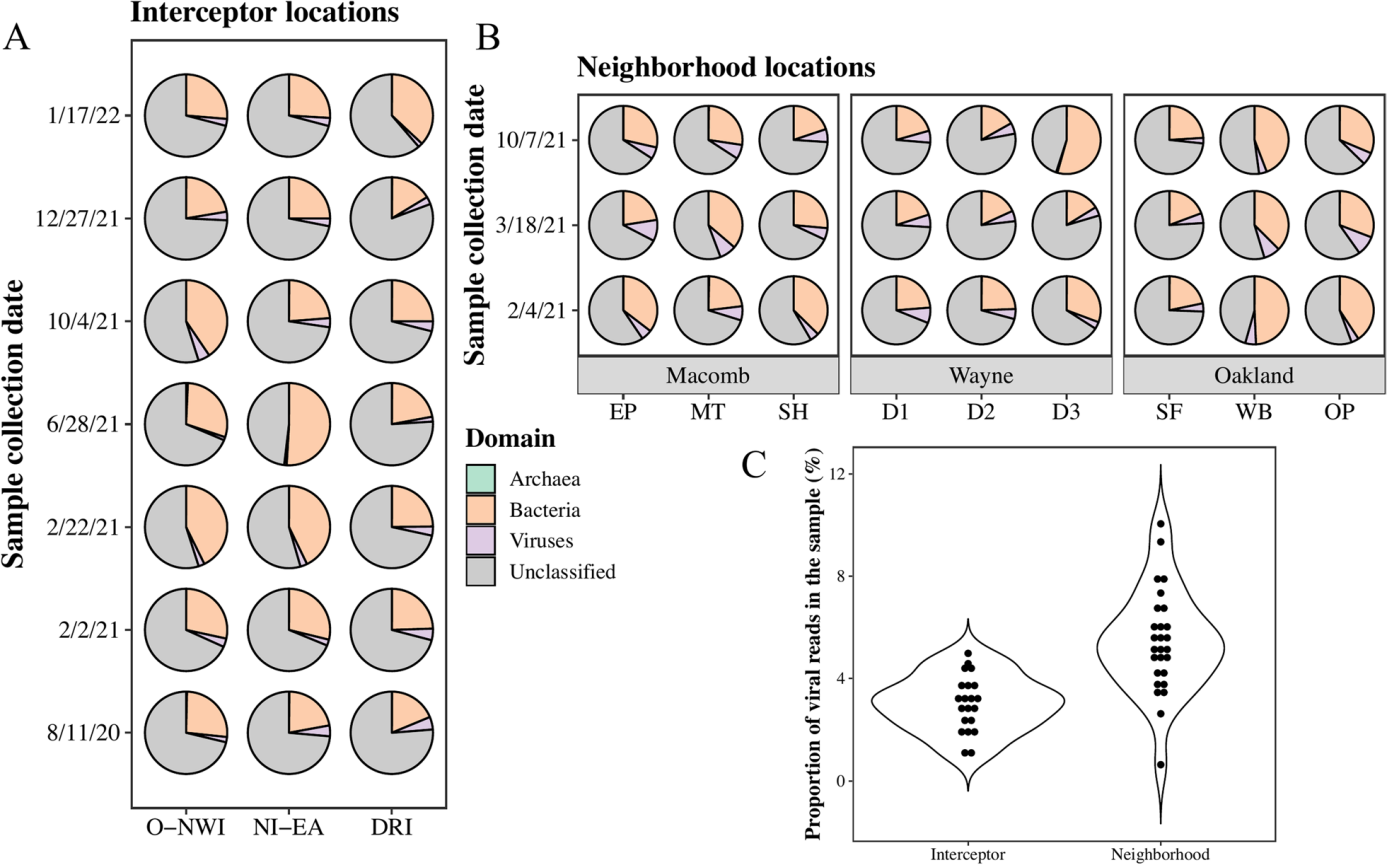
Proportion of viral reads sequenced in study samples. Pie charts indicate the proportion of archaea, bacteria, and viruses in the interceptor (**A**) and neighborhood (**B**) samples. Violin plots show the mean value and data distributions of viral proportions in the interceptor and neighborhood samples (**C**)

A total of 1568.20 GB of sequencing data were obtained from the 48 samples with an average yield of 30.72 GB per sample. There were 4.82 billion clean reads after trimming. The count of clean reads in the wastewater samples ranged from 17.58 million to 123.19 million, with an average count of 95.58 million. Results of the comparison between the clean reads and the NCBI non-redundant database showed that 45.0–80.7% of the reads were unclassified (Fig. 3) and 16.2–54.3% of reads were classified as bacteria. The proportion of the reads that were from viruses ranged from 0.63 to 10.0% (Fig. 3). Deviations of these proportions might be associated with both the variations of wastewater quality characteristics and biases generated in the sample preparation and sequencing steps (e.g., uneven amplification). To improve taxonomy mapping, clean reads were assembled into a total of 90.73 million contigs for the 48 samples using MEGAHIT software [40]. The number of contigs obtained for each sample ranged from 0.24 million to 2.85 million, with an average contig count of 1.79 million.

Contigs were compared against the NCBI’s RefSeq virus database. In total, 3.77 million viral contigs were obtained from the 48 samples. The number of viral contigs per sample ranged from 6593 to 133,335, with an average of 74,163. The proportion of contigs within the sample that were viral ranged from 1.74 to 5.80%. To improve identification of human viruses, assembled contigs were compared with a custom Swiss-Prot human virus protein database. For the 48 samples, the number of human viral contigs per sample ranged from 954 to 9416, with the average number being 5464. The proportion of human viral contigs across the samples ranged from 0.25 to 0.46%, with an average of 0.31%.

### Virus and human virus composition in wastewater samples

Viruses belonging to *Uroviricota* phylum, *Nucleocytoviricota* phylum, and *Phixviricota* phylum were identified in the wastewater samples. Ranges of the proportion of these three phyla in the samples were 66.6–93.6%, 1.40–21.0%, and 0.94–5.59%. Taxonomic composition of viruses at the family level was normalized to the virus population in the wastewater samples and visualized (Additional file 1: Fig. S1). Viral families with a normalized proportion less than 1.00% were categorized as “Other.” As shown in Additional file 1: Fig. S1, contigs affiliated with bacteriophage families *Myoviridae*, *Siphoviridae*, and *Podoviridae* comprised a large proportion of the virus community in wastewater and they belong to the *Uroviricota* phylum. Consistent findings have been reported in previous work [41, 42]. Ranges of the proportion of the viral community for the *Myoviridae*, *Siphoviridae*, and *Podoviridae* families were 31.0–40.1%, 22.1–32.8%, and 7.21–14.8% (Additional file 1: Fig. S1), respectively, and their average proportions were 35.2%, 28.2%, and 11.7%, respectively, across the 48 samples. In addition, notable proportions of viruses belonging to *Nucleocytoviricota* phylum were observed. They were *Mimiviridae* (0.30–5.06%), *Pandoravirus* (0.07–1.79%), *Pithoviridae* (0.04–1.21%), and *Phycodnaviridae* (0.90–11.8%). Viruses in *Mimiviridae*, *Pandoravirus*, and *Pithoviridae* families are the eukaryotic giant viruses that infect the amoebozoan species Acanthamoeba, which is one of the most common protozoa in a variety of environments including the wastewater treatment systems [43]. Members of the *Phycodnaviridae* family can infect a range of protists and algae [44, 45]. Viruses that infect mammals were identified in relatively small proportions. For example, contigs related to the dsDNA *poxvirus* family were found across the 48 samples with a proportion ranging from only 0.18 to 1.78%, with an average of 0.48%. Contigs related to viruses belonging to the *Parvoviridae*, *Herpesviridae*, and *Astroviridae* families were also found at relatively low proportion, all less than 0.1%, and were thus classified as “Other” (Additional file 1: Fig. S1).

**Fig. 4:**
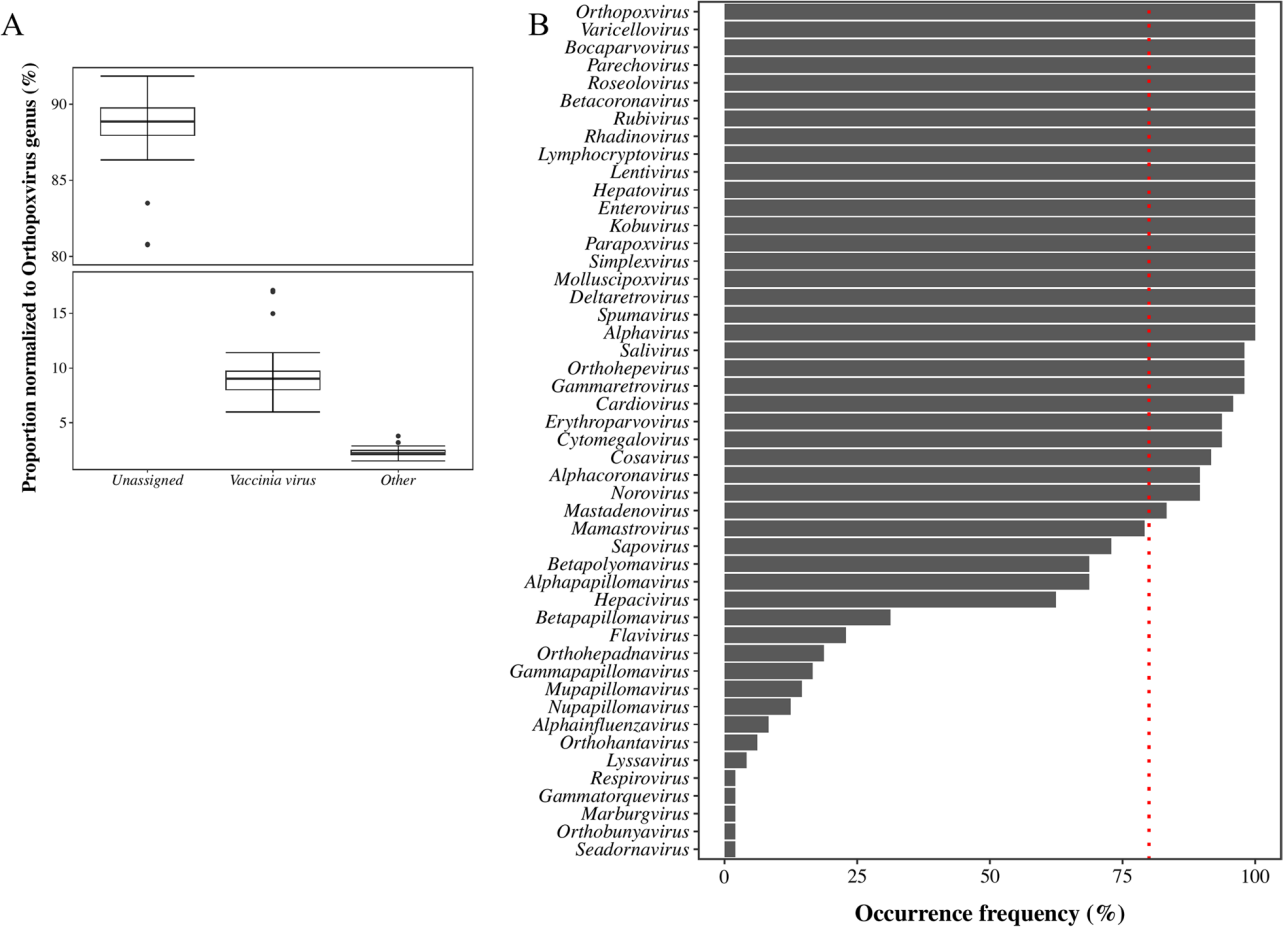
Proportion of *Orthopoxvirus* species (**A**) and human virus occurrence (**B**) in wastewater in the metropolitan Detroit Area, Michigan. Proportion of species identified in the *Orthopoxvirus* genus, with values normalized to the *Orthopoxvirus* population (**A**). Occurrence frequency (%) of each human virus, at the genus level, in the wastewater samples (**B**)

As previously mentioned, to improve human virus discovery, assembled contigs were compared with a custom Swiss-Prot human virus database. Contigs related to a diverse human virus group were identified and classified at the genus level (Additional file 1: Fig. S2). Values were normalized to the human virus population for each sample. Contigs related to viruses belonging to the *Orthopoxvirus* genus were dominant among the 48 samples, with a normalized proportion ranging from 55.9 to 75.1% of the total human virus-related contigs. Further compositional analysis at the species level indicated that approximately 88.5% of *Orthopoxviruses* were unable to be assigned (Fig. 4A). Vaccinia virus (VACV) was the primary genus assigned to *Orthopoxivirus,* with an averaging proportion of 9.17% among only *Orthopoxviruses,* across the 48 samples (Fig. 4A). Following *Orthopoxviruses*, other human viral genera identified by contig as representing a relatively large proportion were *Rhadinovirus*, *Parapoxvirus*, *Varicellovirus*, *Hepatovirus*, *Simplexvirus*, and *Mulluscipoxvirus* (Additional file 1: Fig. S2).

The occurrence frequency of each human virus across the 48 samples was calculated (Fig. 4B). Contigs related to *Orthopoxvirus*, *Varicellovirus, Bocaparvovirus, Parechovirus, Roseolovirus, Betacoronavirus, Rubivirus, Rhadinovirus, Lymphocryptovirus, Lentivirus, Hepatovirus, Enterovirus, Kobuvirus, Parapoxvirus, Simplexvirus, Molluscipoxvirus, Deltaretrovirus, Spumavirus*, and *Alphavirus* genera were detected in all 48 samples. For the *Salivirus, Orthohepevirus, Gammaretrovirus, Cardiovirus, Erythroparvovirus, Cytomegalovirus, Cosavirus, Alphacoronavirus, Norovirus*, and *Mastadenovirus* genera, the occurrence frequency was greater than 80%.

**Fig. 5:**
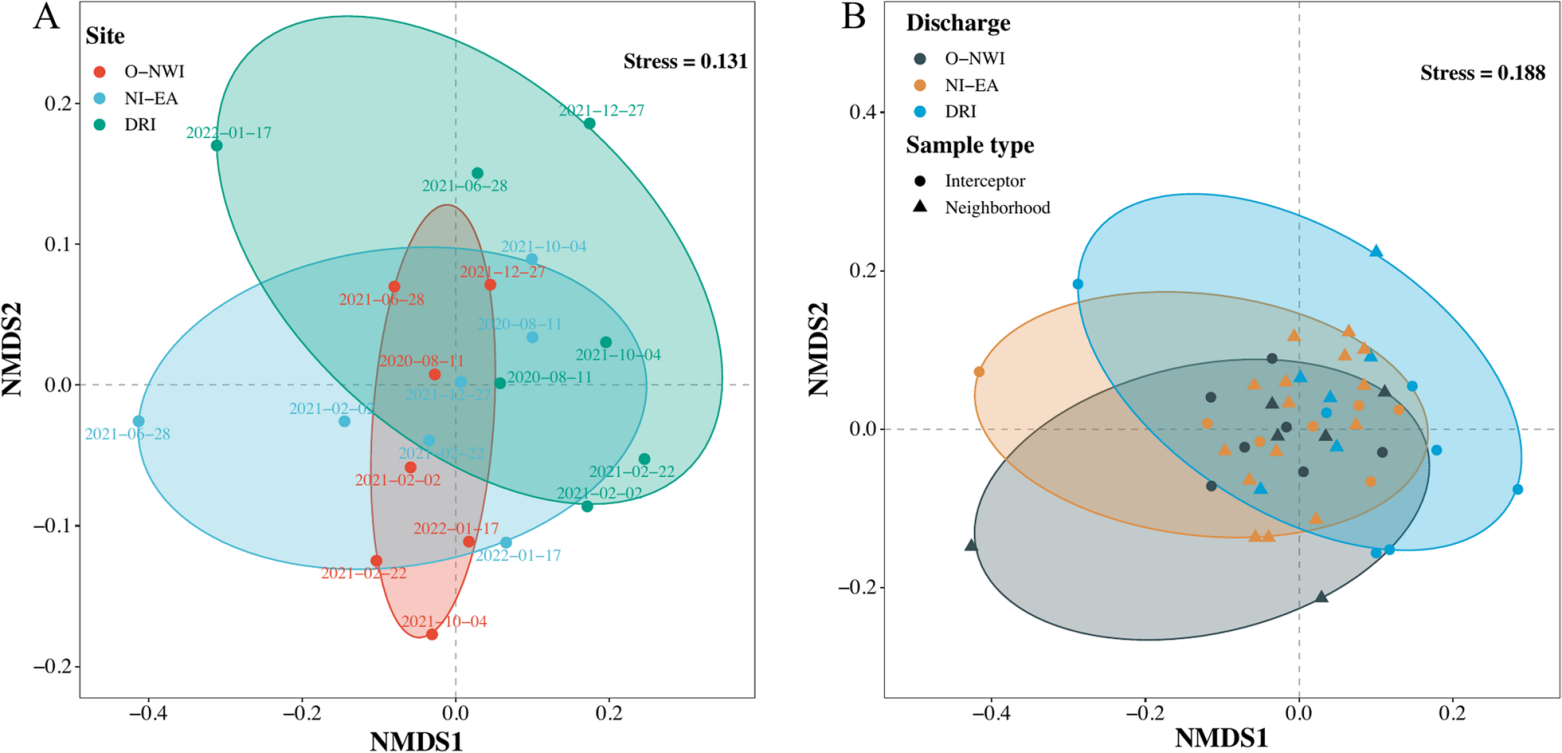
Non-metric multidimensional scaling (NMDS) analysis of human viruses at the genus level. Non-metric multidimensional scaling analysis using the Bray–Curtis dissimilarity method for distance calculation for human viruses in interceptor samples collected from the O-NWI, NI-EA, and DRI interceptors (**A**), and interceptor and neighborhood samples (**B**)

Analysis of NMDS was performed based on human virus composition, to investigate potential spatial or temporal patterns of human virus occurrence. When investigating the wastewater samples collected from the three WRRF interceptors, plots of the NMDS analysis showed a potential spatial pattern (Fig. 5A). A similar pattern was found when neighborhood samples were included and grouped according to the interceptor that they discharge into (Fig. 5B). These results are reasonable since the samples were collected in close proximity and at similar time points.

### Species classification within the *Astrovirus*, *Enterovirus*, *Norovirus* and *Betapolyomavirus* genera

**Fig. 6:**
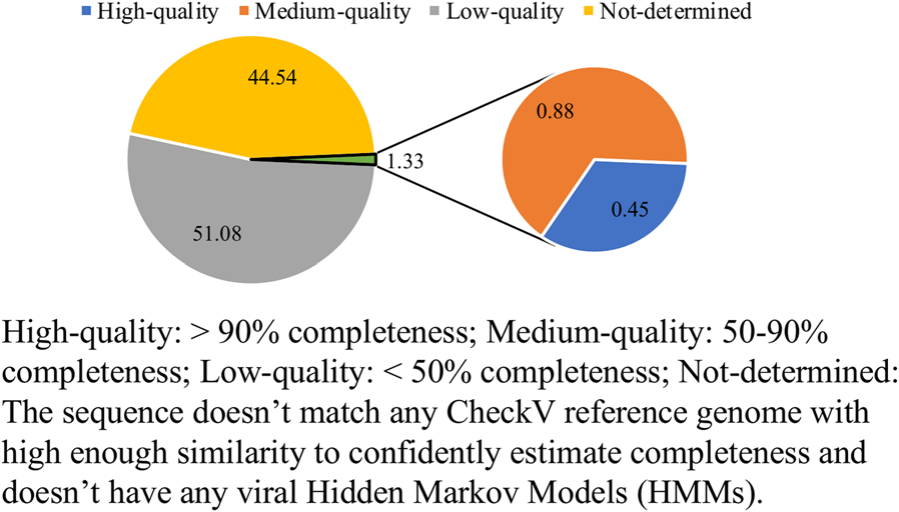
Percentages of quality tiers of the human viral contigs assessed with CheckV. Percentages (%) of human viral contigs assigned to different quality tiers (High-quality, Medium-quality, Low-quality, and Undetermined quality) by assessing the completeness of metagenome-assembled viral contigs with CheckV

**Fig. 7:**
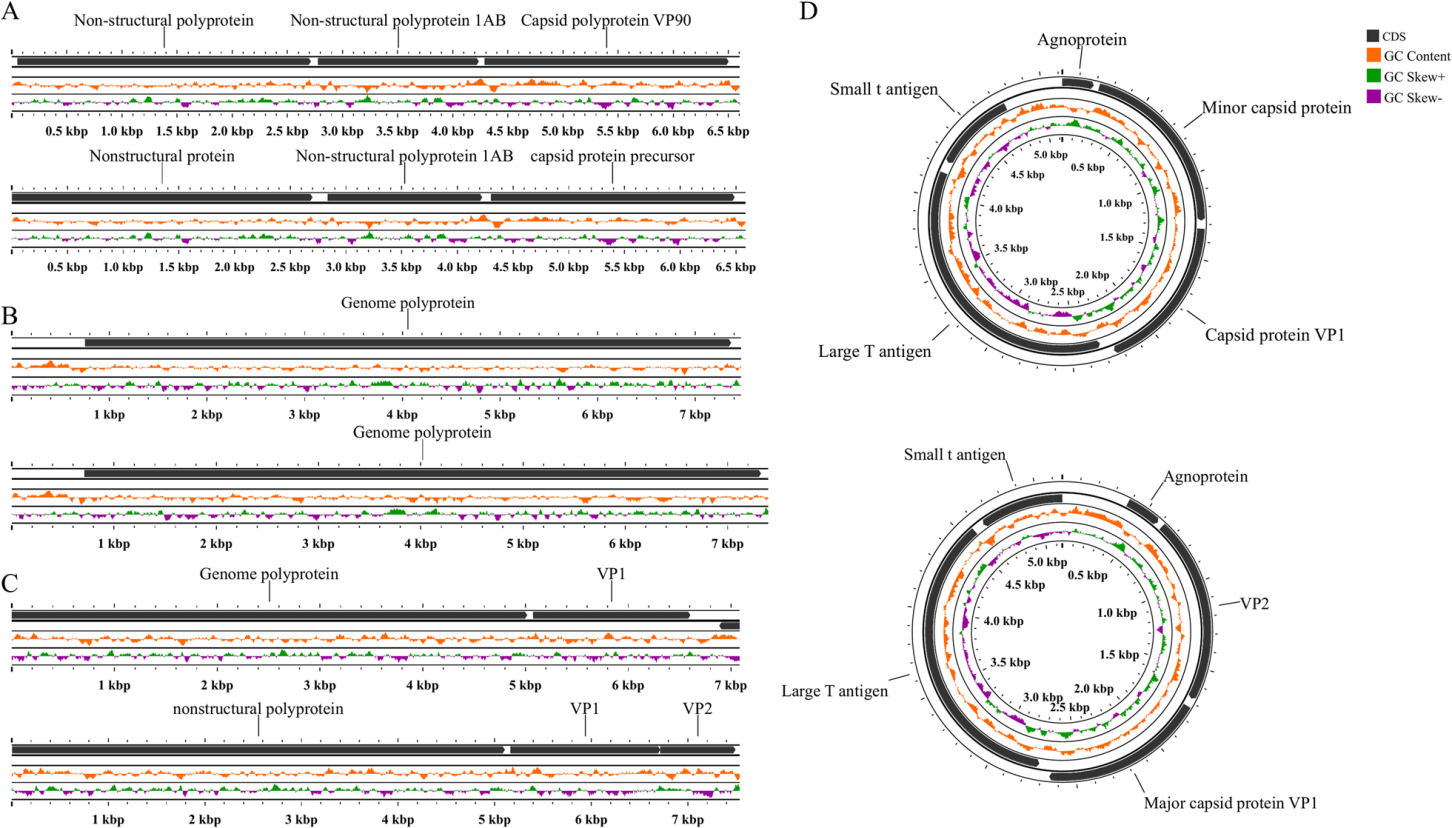
Genome structure of the four nearly complete genomes recovered and their closest reference genomes. **A** *Astrovirus* (top genome: *Astrovirus* genome recovered from sample collected from MT on March 18, 2021; bottom genome: its closest reference genome, *Astrovirus VA1*). **B** *Enterovirus* (top genome: *Enterovirus* genome recovered from sample collected from D1 on March 18, 2021; bottom genome: its closest reference genome, *Human coxsackievirus A1*). **C** *Norovirus* (top genome: *Norovirus* genome recovered from sample collected from D1 on October 7, 2021; Bottom genome: its closest reference genome, *Norovirus GII 2*). **D** *Betapolyomavirus* (top genome: *Betapolyomavirus* genome recovered from sample collected from EP on March 18, 2021; bottom genome: its closest reference genome, *Betapolyomavirus hominis*)

To verify the completeness (A ratio between the contigs length and the length of matched reference in CheckV genome database) [34] of the draft genomes recovered, a quality checking process using the CheckV platform [34] was performed. Percentages of assembled human viral-related contigs assigned to different quality tiers are shown in Fig. 6. High-quality contigs were screened further manually to find the nearly complete human viral genomes. Four contigs affiliated with the *Astrovirus, Enterovirus, Norovirus,* and *Betapolyomavirus* genera were determined to be recovered with a high level of completeness (Table 2). Genomic structures of these four draft genomes and their closest reference genomes are visually represented in Fig. 7. One contig affiliated with the *Mamastrovirus* genera recovered from samples from the MT neighborhood site (collected on 03/18/2021) was identified as being structurally similar to *Astrovirus VA1*, with genome similarity to *Astrovirus VA1* genome as 0.9280 (Table 2). One contig affiliated with the *Enterovirus* genera recovered from sample D1 (collected on 03/18/2021) was identified as being structurally similar to *Human coxsackievirus A1*, with genome similarity to *Human coxsackievirus A1* genome as 0.9644 (Table 2). The closest genome to one of the *Norovirus* contigs recovered from a D1 sample (collected on 10/07/2021) was found to be *Norovirus GII.2*, with genome similarity to *Norovirus GII.2* genome as 0.8635 (Table 2). Similarly, a contig affiliated with genus *Betapolyomavirus* was recovered in a sample collected from the EP neighborhood site and determined to be structurally similar to *Betapolyomavirus hominis,* with genome similarity to *Betapolyomavirus hominis* genome as 0.9003 (Table 2).

**Table 2:** The similarities between the four genomes recovered and their closest reference genomes

Nearly complete genome recovered	Closest reference genome				Similarity of genomes (S_G_)
Classification	Length (nt)	Classification	Length (nt)	Genome Structure	
*Mamastrovirus* (MT.2021.03.18)	6603	*Astrovirus VA1* (NC_013060)	6586	ssRNA (linear)	0.9280
*Betapolyomavirus* (EP.2021.03.18)	5122	*Betapolyomavirus hominis* (NC_001538)	5153	dsDNA (circular)	0.9003
*Enterovirus* (D1.2021.03.18)	7469	*Human coxsackievirus A1* (JX1741)	7398	ssRNA (linear)	0.9644
*Norovirus* (D1.2021.10.07)	7085	*Norovirus GII 2* (NC_039476)	7536	ssRNA (linear)	0.8635

Astroviruses are a well-known causative agent of gastroenteritis in many hosts, including humans. There are eight types of human astroviruses reported in previous studies, among them *Astrovirus VA1*, the genotype commonly discovered from human cases of encephalitis [46, 47]. Genomes of astroviruses range in size from 6.1 to 7.9 kb and contain two nonstructural polyproteins and one capsid protein (Fig. 7A). The high degree of similarity between the *Astrovirus* contig recovered in our study and *Astrovirus VA1* (NC_013060) indicates the possible presence of genotype VA1 in wastewater, and its circulation in the community.

The second nearly complete draft genome was from the *Enterovirus* genus. *Enterovirus C* consists of more than 20 serotypes and its genome is a single-stranded RNA consisting of a long, single open reading frame (ORF) [48]. Length of *Enterovirus* genomes is approximately 7.4 kb. The *Enterovirus* contig recovered in this study was found to be similar to *Human coxsackievirus A1* (CVA1, JX1741) with the genomic similarity being 0.9644 (Fig. 7B). This CVA1 was previously identified in symptomatic individuals (high school students in the USA after a trip to Mexico in 2004) [49].

*Norovirus* is the major pathogen associated with acute gastroenteritis worldwide. Sizes of Norovirus genomes range from 7.5 to 7.7 kb, and consist of three ORFs (Fig. 7C) [50]. Genotypes of *Noroviruses* are diverse and two major groups that affect humans include GI and GII [51]. In North American, levels of human Norovirus GII in wastewater influent were found to be higher than those of GI [52]. In this study, the near-complete contig affiliated with the *Norovirus* genus was found to be most similar to *Norovirus GII.2* (NC_039476), with a genomic similarity of 0.8635. The relatively reduced similarity may be due to the incomplete sequence recovered in the study. The length of reference *Norovirus GII.2* is 7536 nt, while the recovered *Norovirus* contig was 7085 nt. Genomes of *Betapolyomaviruses* consist of circular DNA with lengths of approximately 5 kb [53] (Fig. 7D). The closest reference genome to it was *Betapolyomavirus hominis* (NC_001538), with the genomic similarity being 0.9003.

### Consistent identification of human viruses in wastewater and the associated clinical disease cases in Detroit communities

To understand human virus occurrence in wastewater and its potential connection with observed clinical disease cases in the Detroit metropolitan area, types of human viruses, primary transmission routes, potential diseases the viruses are related to, and reported disease cases in communities from the Detroit metropolitan area are summarized (Table 3). Human viruses that were identified in this study are affiliated with 48 genera. Among these genera, dsDNA *Orthopoxvirus* was found to be the most abundant genus of human viruses. Most of the assigned contigs within the *Orthopoxvirus* genus in this study are classified as vaccinia virus (VACV) (Fig. 4A), consistent with the finding of our previous work, in which VACV was found to be the most prevalent species within the *Orthopoxvirus genus* [12]. Worldwide eradication of smallpox was officially declared in 1980 and the VACV-based vaccine ceased after more than 150 years of successful prevention against smallpox. However, vaccine is still recommended for individuals with unusual potential exposure, such as laboratory workers who handle variola virus [54, 55]. In addition, US military continues to vaccinate against smallpox due to concerns about potential bioterrorism involving stored smallpox virus [56].

**Table 3:** Classification of detected human virus genera, their primary transmission routes, and corresponding human disease in the study area during the sampling years

Family	Genus	Structure	Primary Transmission Route	Associated Human Disease	Confirmed Diseases Cases in metropolitan Detroit, MI		References
					2020	2021	
*Adenoviridae*	*Mastadenovirus*	ds DNA	Respiratory, fecal–oral	Common cold			[59]
*Anelloviridae*	*Gammatorquevirus*	ss DNA	Fecal–oral, parenteral, sexual	May be associated with diseases such as unexplained fever, diabetes, cancer			[60]
*Astroviridae*	*Mamastrovirus*	ss RNA (+)	Fecal–oral	Gastroenteritis			[61]
*Caliciviridae*	*Norovirus*	ss RNA (+)	Fecal–oral	Norovirus, Gastroenteritis	104	62	[62]
	*Sapovirus*	ss RNA (+)	Fecal–oral	Gastroenteritis			[63]
*Coronaviridae*	*Alphacoronavirus*	ss RNA (+)	Respiratory	Common cold			[64]
	*Betacoronavirus*	ss RNA (+)	Respiratory, Zoonosis	SARS, MERS, COVID-19	36,567	729,275	[64]
*Filoviridae*	*Marburgvirus*	ss RNA (-)	Zoonosis, fomite	Viral hemorrhagic fevers			[65]
*Flaviviridae*	*Flavivirus*	ss RNA (+)	Zoonosis, arthropod bite/borne	Encephalitis, dengue fever	8	2	[66]
				West Nile	35	44	
				Zika	4	NR	
	*Hepacivirus*	ss RNA (+)	Sexual, blood	Hepatitis C	2541	1739	[67]
*Hantaviridae*	*Orthohantavirus*	ss RNA (-)	Zoonosis, urine, saliva	Viral hemorrhagic fevers, hantavirus pulmonary syndrome			[68]
*Hepeviridae*	*Orthohepevirus*	ss RNA (+)	Fecal–oral	Hepatitis E	5	7	[69]
*Hepadnaviridae*	*Orthohepadnavirus*	RT	Sexual contact, blood	Hepatitis B	3066	376	[70]
*Herpesviridae*	*Rhadinovirus*	ds DNA	Sexual contact, saliva	Skin lymphoma			[71]
	*Varicellovirus*	ds DNA	Respiratory, contact	Chickenpox	62	60	[72]
				Shingles	386	244	
	*Simplexvirus*	ds DNA	Sexual contact, saliva	Skin lesions			[73]
	*Roseolovirus*	ds DNA	Respiratory, contact	Encephalitis			[74]
	*Lymphocryptovirus*	ds DNA	Zoonosis, animal bite, contact, saliva	Encephalitis, mononucleosis			[75]
	*Cytomegalovirus*	ds DNA	Contact, urine, saliva	Mononucleosis, pneumonia			[76]
*Matonaviridae*	*Rubivirus*	ss RNA (+)	Respiratory	Rubella	4	3	[77]
*Orthomyxoviridae*	*Alphainfluenzavirus*	ss RNA (-)	Respiratory, zoonosis, animal contact	Acute febrile respiratory tract infection			[59]
*Paramyxoviridae*	*Respirovirus*	ss RNA (-)	Respiratory	Common cold			[59]
*Papillomaviridae*	*Alphapapillomavirus*	ds DNA	Sexual, contact	Genital warts, cervical cancer, skin warts			[78]
	*Nupapillomavirus*	ds DNA	Contact	Skin warts			[78]
	*Betapapillomavirus*	ds DNA	Contact	Warts, papilloma, malignant tumors			[78]
	*Gammapapillomavirus*	ds DNA	Contact	Warts, papilloma			[78]
	*Mupapillomavirus*	ds DNA	Contact	Skin warts			[78]
*Parvoviridae*	*Bocaparvovirus*	ss DNA	Respiratory	Acute respiratory illness			[79]
	*Erythroparvovirus*	ss DNA	Respiratory	Fifth disease, skin lesions			[80]
*Peribunyaviridae*	*Orthobunyavirus*	ss RNA (-)	Zoonosis, arthropod bite	Encephalitis, fever, joint pain			[81]
*Picornaviridae*	*Hepatovirus*	ss RNA (+)	Fecal–oral	Hepatitis A	13	15	[82]
	*Parechovirus*	ss RNA (+)	Respiratory	Mild, gastrointestinal, or respiratory illness			[83]
	*Enterovirus*	ss RNA (+)	Fecal–oral, respiratory	Meningitis, myocarditis, paralysis, common cold, diarrhea, neurological disorder, poliomyelitis			[84]
	*Cardiovirus*	ss RNA (+)	Zoonosis	Encephalitis			[85]
	*Kobuvirus*	ss RNA (+)	Fecal–oral	Gastroenteritis			[86]
	*Salivirus*	ss RNA (+)	Fecal–oral	Gastroenteritis			[87]
	*Cosavirus*	ss RNA (+)	Fecal–oral (probable)	Gastroenteritis (probable)			[88]
*Polyomaviridae*	*Betapolyomavirus*	ds DNA	Respiratory	May be associated with central nervous system disease			[89, 90]
*Poxviridae*	*Orthopoxvirus*	ds DNA	Respiratory, zoonosis, contact	Skin lesions, smallpox			[54]
	*Parapoxvirus*	ds DNA	Zoonosis, contact	Skin lesions			[91]
	*Molluscipoxvirus*	ds DNA	Contact	Skin lesions			[92]
*Reoviridae*	*Seadornavirus*	dsRNA	Zoonosis, arthropod bite	Encephalitis			[93]
*Retroviridae*	*Deltaretrovirus*	RT	Sexual contact, maternal-neonatal	Leukemia			[94]
	*Spumavirus*	RT	Animal bites	Associated with T lymphocyte differentiation and monocyte activation			[95]
	*Gammaretrovirus*	RT	Often from mother to offspring	Multiple sclerosis (probable)			[96]
	*Lentivirus*	RT	Sexual contact, blood	HIV	334	412	[97]
*Rhabdoviridae*	*Lyssavirus*	ss RNA (-)	Zoonosis, animal bite	Fatal encephalitis, encephalitis			[98]
*Togaviridae*	*Alphavirus*	ss RNA (+)	Zoonosis, arthropod bite	Fever, joint pain, chikungunya, encephalitis, pogosta disease	2	1	[99]

Blank cells indicate that data is not available. Human diseases in red font correspond to the number of confirmed cases listed in the following column. Disease cases were based on the weekly disease reports published by the Michigan Disease Surveillance System (MDSS) in years 2021 and 2022. Abbreviations: ds DNA: double stranded DNA; ss DNA: single-stranded DNA; ss RNA (+): single-stranded positive RNA; ss RNA (−): single-stranded negative RNA; RT: reverse transcribing viruses. SARS: severe acute respiratory syndrome; MERS: Middle Eastern respiratory syndrome; COVID-19: coronavirus disease 2019

*Betacoronaviruses* include two common human coronaviruses (OC43 and HKU1) that cause middle east respiratory syndrome (MERS-CoV), severe acute respiratory syndrome (SARS-CoV), and the novel coronavirus that causes coronavirus disease 2019 (SARS-CoV-2). *Betacoronaviruses* were identified in the wastewater samples (Figs. S2 and 4B), as expected, since samples were collected during the COVID-19 pandemic period. These samples have been analyzed with ddPCR, and SARS-CoV-2 occurrence has been confirmed [6, 7, 22].

Corresponding to the presence of *Varicellovirus* (varicella-zoster virus, VZV, or HHV-3) in the sampled wastewater, clinical cases of chickenpox and shingles in the Detroit metropolitan area in 2020 numbered 62 and 386, respectively, and in 2021, numbered 60 and 244, respectively [57, 58]. Contigs assigned to *Roseolovirus* were found in all of the samples (Fig. 4B), indicating potential human infection by these pathogens in communities. Contigs affiliated with *Rubivirus* were found frequently in this study (Fig. 4B) and cases of rubella reported in 2020 and 2021 equal 4 and 3 in the study area [57, 58]. *Bocaparvovirus* and *Erythroparvovirus*, two respiratory human viruses belonging to the *Parvoviridae* family, were prevalent in all of the samples (Fig. 4B). *Parecovirus* related contigs, which may be associated with *human parechovirus* (HPeV), was also found frequently in the samples (Fig. 4B). Infections of HPeV are reported to be associated with some mild respiratory and gastrointestinal diseases, but can also cause serious disease such as meningitis, encephalitis, and neonatal sepsis [83].

Beyond contigs related to respiratory viruses, contigs related to viruses potentially transmitted through a fecal–oral route were also detected with high frequency in our samples (Fig. 4B). These include *Mamastrovirus, Norovirus, Orthohepevirus, Hepatovirus, Enterovirus, Kobuvirus, Salivirus,* and *Cosavirus* related contigs (Fig. 4B). Contigs related to the *Mamastrovirus* genus were found in 38 samples. Species within the *Mamastrovirus* genus are reported to commonly cause symptoms such as mild diarrhea, as well as less commonly, vomiting, headache, and fever [61]. *Norovirus* has been responsible for acute non-bacterial gastroenteritis diseases worldwide, for decades [62] and were found in 90% (43/48) of the 48 samples taken. In the Detroit metropolitan area, *Norovirus* disease cases reported were 104 in 2020 and 62 in 2021 [57, 58]. The *Orthohepevirus* genus contains the well-known causative viral pathogen, *hepatitis E virus* (HEV); contigs affiliated with the genus were found in 47 of 48 wastewater samples. In 2020 and 2021, there were five and seven hepatitis E cases reported in the Detroit metropolitan area [57, 58].

The *Hepatovirus* genus contains another common hepatitis virus: hepatitis A (also known as *Hepatovirus A* virus, HAV) [82]. Contigs related to it were identified in all of the collected samples (Fig. 4B). There were 13 and 15 hepatitis A cases reported in 2020 and 2021, respectively, in the study area [57, 58]. The *Enterovirus* genus includes various viral pathogens that can cause diseases with symptoms ranging from mild symptom to the disabling and sometimes life-threatening disease of paralytic poliomyelitis. *Enterovirus D68* (EV-D68) is a well-known non-polio enterovirus that can cause respiratory illness. Contigs related to the *Enterovirus* genus were identified in all of the collected samples (Fig. 4B). Like *Hepatovirus* and *Enterovirus*, *Kobuvirus, Salivirus* and *Cosavirus* also belong to the *Picornavirus*. Occurrence frequencies of these three viruses were higher than 90% in this study (Fig. 4B). These viruses are often associated with causing diarrhea and gastroenteritis [86, 88, 100].

Other than respiratory and fecal–oral transmission, viruses transmitted by blood and other bodily fluids can cause health concerns. The genus *Lentivirus* was detected in all 48 samples (Fig. 4B). Human immunodeficiency viruses (HIV), which belong to the *Lentivirus* genus, attack the body’s immune system and may lead to acquired immunodeficiency syndrome (AIDS). Through a most recent HIV statistics published by CDC, an estimated 1.2 million people in the USA and dependent areas had HIV at the end of 2021, about 87% of these people knew they had HIV [101]. Within the Detroit metropolitan area during the sampling years of 2020 and 2021, 334 and 412 HIV cases were reported, respectively [57, 58]. Linking the presence of genus *Lentivirus* in wastewater and the disease cases in the Detroit community is difficult, due to the limited information of species composition within this genus and the incidence rates for HIV in the community.

The genus *Orthohepadnavirus*, which contains the *hepatitis B virus* (HBV), was present in 19% (9/48) of the wastewater samples (Fig. 4B). The total number of *hepatitis B* cases reported in the Detroit metropolitan area in 2020 and 2021 were 3066 and 376, respectively [57, 58]. Through the 2021 viral hepatitis surveillance report, a decrease of viral hepatitis cases in 2020 and 2021 in the USA was reported; however, this should be interpreted with caution, since it may be related to fewer people being tested for viral hepatitis during the COVID-19 pandemic [102]. The *Hepacivirus* genus, which contains the *hepatitis C virus* (HCV), was present in 63% (30/48) of the wastewater samples collected in this study (Fig. 4B). Cases of hepatitis C reported in the Detroit metropolitan area in 2020 and 2021 were 2541 and 1739, respectively [57, 58]. The *Lymphocryptovirus* genus, which includes the human-infecting *human gammaherpesvirus 4* (Epstein–Barr virus, EBV) transmitted through bodily fluids, was detected in all of the 48 samples collected (Fig. 4B).

There are other viral pathogens which are transmitted to humans through arthropod vectors, like mosquitoes and ticks. For example, the genus *Flavivirus* was detected in 23% (11/48) of the wastewater samples in this study (Fig. 4B). Within genus *Flavivirus*, mosquito-borne viruses include yellow fever virus, dengue fever virus, Japanese encephalitis, West Nile viruses, and zika virus. There were eight and two cases of dengue fever reported in the study area in years 2020 and 2021, respectively, 35 and 44 cases of West Nile disease reported in years 2020 and 2021, respectively, and four and zero cases of Zika disease reported in 2020, and 2021, respectively [57, 58]. The genus *Alphavirus* consists of infectious viruses that cause eastern equine encephalitis (eastern equine encephalitis virus [EEEV]), and Chikungunya (chikungunya virus [CHIKV]). Contigs assigned to the genus *Alphavirus* were detected in all of the 48 samples. In 2019, the largest outbreak of eastern equine encephalitis (EEE) ever recorded in Michigan was observed, with 10 human cases (6 fatal). In 2020, an outbreak of EEE of 4 human cases occurred in Michigan [103, 104]. There were two and one cases of Chikungunya reported in the Detroit metropolitan area in the years of 2020 and 2021, respectively [57, 58].

Overall, the detected genera in wastewater are only an indication of potential presence of associated viruses in the population. The investigation approach needs to be further optimized and collaboration between environmental researchers, public health officials, and epidemiologists needs to be strengthened to maximize the application of the wastewater-related data.

## Discussion

Respiratory viruses were found to be prevalent in the wastewater samples examined in this work, which is interesting since typically wastewater surveillance is thought as a tool to investigate mainly the waterborne or fecal–oral transmitting human viruses [105]. Wastewater monitoring of respiratory viruses started from 2009 and has grown rapidly as highlighted by the success of wastewater surveillance of SARS-CoV-2 [106]. In a proof-of-concept study, multiple respiratory viruses (e.g., *Bocavirus*, *Parechovirus*, *Rhinovirus A*, and *Rhinovirus B*) were detected in wastewater samples from four wastewater treatment plants in Queensland, Australia [106]. A range of respiratory virus concentrations in wastewater were characterized and analyzed to link virus concentrations in wastewater to disease cases in the community [107]. In our study, respiratory viruses including *Bocaparvovirus*, *Betacoronavirus*, *Rubivirus*, and *Erythroparvovirus* were identified. These indicate that surveillance of respiratory viruses in wastewater could be a reliable tool to inform the presence or trends of infectious diseases associated with the respiratory virus circulation in a community.

It is reported that around 75% of the emerging infectious diseases have a zoonotic origin and through the host-virus interactions analyses, rodents and bats are among the major reservoirs of zoonotic viruses [108]. In this work, numerous potentially zoonotic viruses were detected in wastewater in metro Detroit area. Viral contigs related to *Parapoxvious*, *Simplexvirus*, *Molluscipoxvirus*, *Deltaretrovirus*, and *Spumavirus*, which are potentially associated with zoonotic diseases, were identified in all of the 48 samples. However, relationships of zoonotic viruses and the associated disease cases in a given community remain unclear. By longitudinally monitoring the hepatitis E and rat hepatitis E in wastewater in Cordoba, Spain from March 2021 to March 2023, Maria et al. evaluated the possible correlation between the detection of hepatitis E and rat hepatitis E in wastewater and their clinical cases[109], no correlation was observed through their dataset. Further studies are needed to address the relationship between zoonotic viruses in wastewater and clinical disease in urban and rural settings.

Consistent identification of human viruses in wastewater and the associated disease cases in clinical data highlights the potential application of wastewater surveillance for identifying human virus occurrence in a given community. Constructing relationships between human viruses in wastewater and clinically confirmed cases could be challenging, but beneficial for disease control. During the COVID-19 pandemic, both comprehensive wastewater surveillance data of SARS-CoV-2 and clinical data were collected and modeled. Predictive intelligence methods have been developed, showing that early warning of disease surges can be created by correlating wastewater data with clinical data [6]. Signals from sequencing data were correlated to the clinical disease cases in a wastewater surveillance study in Houston and El Paso in Texas. To be specific, the reads per kilobase of transcript per million filtered reads (RPKMF) was used to reflect the relative virus levels (i.e., SRAS-CoV-2) in a given sample [110]. However, quantification of viruses using sequencing and metagenomics approaches is challenging. If a virus of potential concern is detected during diversity screening using metagenomics, follow-up testing with conventional methods such as ddPCR is recommended for quantification.

The approach described in this paper is promising. However, it is important to note that human viruses in wastewater are diverse and vary in their morphology, transmission pathway, and pathogenesis, making it challenging to detect them all and relate their presence in wastewater to the clinical cases reported in the community. Limitations of this study are summarized as follows:

Firstly, the untargeted sequencing approach is not sensitive enough to identify human viruses at a fine taxonomic level in wastewater, which is necessary to relate to the diseases circulating in a given community. The presented findings in wastewater samples are primarily at the genus level. Viral pathogen analysis at the strain or genotype level will help researchers to understand infection and outbreak patterns in communities and will provide insights into disease control and prevention. Comprehensive surveillance of specific human virus species is necessary to understand the epidemiology and potential virulence of outbreaks [47, 48, 111]. For the determination of specific infectious agents, complete genomic sequences are desirable to assessing viral pathogen threats [112, 113]. Nevertheless, only a few near-complete draft genomes of human viruses have been identified with untargeted metagenomics in this work. Following screening with untargeted metagenomics, targeted capture-based sequencing approaches will be beneficial. Targeted capture-based sequencing has been applied in human infectious disease studies [114] and recently in the wastewater surveillance field [110]. The development of a targeted enrichment methodology as well as deep sequencing methodology will enable findings of human viruses at a fine level and an improved genome coverage, which may offer sensitive and suitable estimation of human viruses in circulation and the possibility of species or variant frequency investigation. Secondly, amplification is usually required in viral sequencing studies to ensure the sufficient nucleic acids needed; we used a random amplification approach in this study. The effects of different amplification methods on human virus discovery need to be assessed. Thirdly, collection of disease case data in a given community is often constrained by resources, human behavior changes, and other parameters. Long-term clinical data are difficult to collect and most often are not available at all for non-reportable diseases. In this work, we used public health records for clinical datasets in the metropolitan Detroit Area in Michigan, which is an area with varied population demographics and human behaviors.

## Conclusions

- Assembled contigs related to diverse human virus genera were detected in raw wastewater samples from the Detroit metropolitan area during the COVID-19 pandemic. In addition to *Betacoronavirus,* detected viruses included *Orthopoxvirus*, *Rhadinovirus, Parapoxvirus*, *Varicellovirus, Hepatovirus, Simplexvirus, Bocaparvovirus, Molluscipoxvirus, Parechovirus, Roseolovirus, Lymphocryptovirus, Alphavirus, Spumavirus, Lentivirus, Deltaretrovirus, Enterovirus, Kobuvirus, Gammaretrovirus, Cardiovirus, Erythroparvovirus, Salivirus, Rubivirus, Orthohepevirus, Cytomegalovirus, Norovirus,* and *Mamastrovirus*. Identification of virus-related contigs using bioinformatic methods should be used as a “screening” tool that will indicate the need for further testing.
- Nearly complete draft genomes of *Astrovirus, Betapolyomavirus, Norovirus,* and *Enterovirus* were recovered in a few of the collected 48 samples, showing that this method can pinpoint circulating pathogens at the species or genotype level. However, targeted sequencing is still required to investigate the spatial and/or temporal pattern of many pathogens at a finer resolution.
- The presence of some human viruses in wastewater was associated with reported clinical disease cases in the community. Some of the detected viral-related sequences belonged to human viruses that are not reported by the local health department. Understanding the relationships between the occurrence and abundance of human viruses in wastewater and associated diseases circulating in the community will require more evidence regarding mechanisms of pathogenesis, transmission of human viruses into the human body, and the potential symptoms of diseases.

### Supplementary Information

**Additional file 1. **Provides method, table and figures addressing: **S1** Library preparation for whole metagenome shotgun sequencing. **S2** Custom of the human-associated virus protein database. **Table S1** Parameters applied in the downstream bioinformatic steps. **Figure S1** Viral families identified in wastewater samples in the Detroit, MI metropolitan area. All values were normalized to virus composition. Families with proportions of less than 1% across all samples were classified as “other”. **Figure S2** Proportion of each human virus genus normalized to the human viruses identified in wastewater. Values were normalized to the human virus composition. Symbol “X” indicates that the virus is not identified in the sample.

## Data Availability

The clinical data of metropolitan Detroit MI community during the sampling years were shown in the main text. The datasets used and/or analyzed during the current study are available from the corresponding author on reasonable request.

## Abbreviations

SARS-CoV-2: Severe acute respiratory syndrome coronavirus 2
WRRF: Water Resource Recovery Facility
GLWA: Great Lakes Water Authority
DRI: Detroit River Interceptor
NI-EA: The North Interceptor-East Arm
O-NWI: The Oakwood-Northwest-Wayne County Interceptor
US EPA: US Environmental Protection Agency
NCBI: National Center for Biotechnology Information
NMDS: Non-metric multidimensional scaling
VACV: *Vaccinia virus*
VZV: *Varicella-zoster virus*
HPeV: *Human parechovirus*
HEV: *Hepatitis E virus*
HAV: *Hepatovirus A virus*
HIV: *Human immunodeficiency virus*
HBV: *Hepatitis B virus*
HCV: *Hepatitis C virus*
EBV: *Epstein–Barr virus*
EEEV: *Eastern equine encephalitis virus*
CHIKV: *Chikungunya virus*
AIDS: Acquired immunodeficiency syndrome
RPKMF: Reads per kilobase of transcript per million filtered reads
