# Supplementary material for: A broad wastewater screening and clinical data surveillance for virus-related diseases in the metropolitan Detroit area in Michigan
Source: Hum Genomics. 2024 Feb 6;18:14. doi: 10.1186/s40246-024-00581-0 (PMC10845806; doi:10.1186/s40246-024-00581-0)
Supplement: Supplementary file 1 — Additional file 1. Provides method, table and figures addressing: S1 Library preparation for whole metagenome shotgun sequencing. S2 Custom of the human-associated virus protein database. Table S1 Parameters applied in the downstream bioinformatic steps. Figure S1 Viral families identified in wastewater samples in the Detroit, MI metropolitan area. All values were normalized to virus composition. Families with proportions of less than 1% across all samples were classified as “other”. Figure S2 Proportion of each human virus genus normalized to the human viruses identified in wastewater. Values were normalized to the human virus composition. Symbol “X” indicates that the virus is not identified in the sample. [file 40246_2024_581_MOESM1_ESM.docx]

**Supplementary Materials**

**A broad wastewater screening and clinical data surveillance for virus-related diseases in the metropolitan Detroit Area in Michigan**

Yabing Li^1^, Brijen Miyani^1^, Russell A. Faust^2^, Randy E. David^3^, Irene Xagoraraki^1^*

^1^Department of Civil and Environmental Engineering, Michigan State University, 1449 Engineering Research Ct, East Lansing, MI 48823, USA

^2^Oakland County Health Division, 1200 Telegraph Rd, Pontiac, MI 48341, USA

^3^Wayne State University, School of Medicine, Detroit MI 48282, USA

*Corresponding author. E-mail address: [xagorara@msu.edu](mailto:xagorara@msu.edu) (I. Xagoraraki)

The supplementary materials provide method, table and figures addressing: **S1** Library preparation for whole metagenome shotgun sequencing. **S2** Custom of the human-associated virus protein database. **Table S1** Parameters applied in the downstream bioinformatic steps. **Figure S1** Viral families identified in wastewater samples in the Detroit, MI metropolitan area.

All values were normalized to virus composition. Families with proportions of less than 1% across all samples were classified as “other”. **Figure S2** Proportion of each human virus genus normalized to the human viruses identified in wastewater. Values were normalized to the human virus composition. Symbol “X” indicates that the virus is not identified in the sample.

**S1 Library preparation for whole metagenome shotgun sequencing**

Libraries were prepared using the Illumina TruSeq Nano DNA Library Preparation Kit with IDT for Illumina – TruSeq DNA Unique Dual Indexes. Completed libraries were checked for quality and quantified using a combination of Qubit dsDNA HS and Agilent 4200 TapeStation HS DNA1000 assays. Libraries were combined on an equimoloar basis into two separate pools of 27 libraries each. Pools were quantified using the Invitrogen Collibri Quantification qPCR kit. Each pool was loaded onto one lane of a NovaSeq S4 flow cell and sequencing was performed in a 2x150 bp paired end format using a NovaSeq 6000 v1.5 300 cycle reagent kit. Base calling was done by Illumina Real Time Analysis (RTA) v3.4.4 and output of RTA was demultiplexed and converted to FastQ format with Illumina Bcl2fastq (v2.20.0).

**S2 Custom of the human-associated virus protein database**

The database was customed from Swiss-Prot database, which is the reviewed section of UniPort Knowledgebase (UniProtKB), a comprehensive, non-redundant, and one of the most widely used protein sequence knowledgebase in the world (Consortium, 2022). The human virus protein database was customed from UniProtKB by using the query builder. To be specific, select search in UniProtKB and click “Advanced” to open the query builder. Firstly, select “Taxonomy” from the dropdown menu and type 10239 for viruses; secondly, select “Virus host” from the dropdown menu and type 9606 for human sapiens; thirdly, select the “Reviewed” and set it as “Yes”. Click on the search button and 6,149 results were retrieved on December 20, 2022. Both fasta and excel formats for the results were obtained.

**Table S1** Parameters applied in the downstream bioinformatic steps.

| **Parameters** | **Notes** |
| --- | --- |
| Q score in Illumina sequencing | The sequencing quality score of the given base; Q30 is considered a benchmark for quality in next-generation sequencing; In this study, for each sample, Q scores of more than 90% R1 reads are greater than 30, and for R2, Q scores of more than 88% reads are greater than 30 |
| Trimming parameters | LEADING: quality; the minimum quality required to keep a base.  TRAILING: quality; the minimum quality required to keep a base.  SLIDINGWINDOW: window size: required quality; window size: the number of bases to average across, required quality: the average quality required.  MINLEN: length; the minimum length of reads to be kept.  In this study, the parameters were decided as below:  LEADING:3 TRAILING:3 SLIDINGWINDOW:4:15 MINLEG:35 |
| E-value selection for the alignments | The Expect value (E) is a parameter that describes the number of hits one can “expect” to see by chance when searching a database of a particular size. The lower the E-value the more “significant” the match is. If E is between 0.01 and 10 ^- 50^, the match can be considered a result of homology.  In this study, two E-values were selected regarding to the different sizes of the databases.  Alignment against the relatively large database: E-value: 10^-3^  Alignment against the custom human virus database E-value: 10^-5^ |
| Parameters when assigning taxonomy with MEGAN | The lowest common ancestor (LCA) assignment algorithm for long reads was applied for taxonomic classification. Top 10 percent of the alignments with a minimum bit score of 50 and contig coverage of at least 80% were included in studying the taxonomic content. |

**Figure S1**

**Figure S2**
